# Body social models of disability: Examining enactive and ecological approaches

**DOI:** 10.3389/fpsyg.2023.1128772

**Published:** 2023-03-08

**Authors:** Alan Jurgens

**Affiliations:** School of Humanities and Social Inquiry, Philosophy Department, University of Wollongong, Wollongong, NSW, Australia

**Keywords:** disability, autism, enactivism, ecological psychology, ecology

## Abstract

Autistic philosopher and neurodiversity proponent Robert Chapman (2021) argues that disability may be best understood by utilizing an ecological functional model where the focus is on the intersection and overlaps between relational contributions to collectives and group functioning with individual functionality. This presents an alternative to both social-relational models of disability advocated by other neurodiversity proponents and the orthodox medical model of disability. While enactivists such as Michelle Maiese and Juan Toro, Julian Kiverstein and Erik Rietveld have also offered relational models of disability that challenge the orthodox medical model, I argue that unlike the ecological functional model, these enactivist models remain problematically committed to an individualist methodology. Drawing on what Miriam Kyselo has labeled the body social problem, I show that the enactivist models not only face theoretical issues, but also practical issues in terms of their recommended intervention strategies for disability. I argue that for these reasons, if enactivists want a relational model of disability, then they should adopt both a neurodiversity paradigm approach and Chapman’s ecological functional model.

## 1. Introduction

Currently, there are numerous competing models of disability that not only provide particular conceptions of the notion of “disability,” but also offer different means for determining how disabilities impact individuals’ wellbeing, when a disability should be considered pathological or a disorder, and set a theoretical basis for practical interventions to improve the wellbeing of disabled individuals. While clinicians typically utilize subtle distinctions to classify various forms of disability such as sensory differences (e.g., deafness), neurological differences (e.g., cerebral palsy), neurodiversity (e.g., autism), etc., my aim here is to examine the theoretical foundations of recent relational models of disability (Toro et al., 2020; Chapman, 2021; Maiese, 2021) in order to determine whether there are any conceptual issues within these models. As such, outside of the context of presenting any particular model’s conception of disability, my use of the term “disability” follows Hoffman (2017) in using it as a neutral umbrella term that covers a wide range of phenomena.^1^

In regard to models of disability, the standard orthodox approach utilized for conceptualizing, investigating, diagnosing, and intervening on disability is the medical model (Boorse, 1975, 2010), which forms the basis for the definitions of many conditions listed in the Diagnostic and Statistical Manual of Mental Disorders (DSM-5). As Chapman (2021, p. 1360) reminds us, within the DSM-5, the harm associated with a mental disorder is described as a “clinically significant disturbance” associated with disability or distress, and is assumed to arise from “a dysfunction in the psychological, biological, or developmental processes” (American Psychiatric Association, 2013, p. 20). As such, a medical model approach views physical or cognitive differences as disabilities, which are also classified as functional deficits, or dysfunctions, that an individual either has or does not have. Since differences are conceived of as deficits to be corrected, the intervention strategies stemming from this model are primarily directed at disabled individuals. In this sense, disability is directly associated with deficit or dysfunction in what critics (Blume, 1998; Singer, 1999; Chown and Beavan, 2011; Armstrong, 2015; Chapman, 2019a, 2021) have labeled as a default pathologization of disability and neurodivergence as disability is determined as divergence from normal functioning.

These critics, working from a neurodiversity paradigm, which began as a social-justice movement, but has transformed into an approach for research, challenge the medical model on the grounds that it is partly responsible for the creation of systemic barriers and negative stigmas the neurodivergent regularly face. Instead, neurodiversity proponents conceive of these differences as simply “manifestations of humanity’s ‘natural variation’ (Jaarsma and Welin, 2012) or ‘dispositional diversity’ (Milton, 2017)” that need to be accepted and accommodated as opposed to corrected or fixed (Chapman, 2021, pp. 1360–1,361).^2^ In sharing this concern for the pathologization of difference, in this article, I set aside concerns with the medical model and instead examine three different relational models of disability (Toro et al., 2020; Chapman, 2021; Maiese, 2021), all of which challenge standard orthodox approaches toward the study of minds, in order to determine what kinds of foundational theoretical commitments can lead to the best possible practical outcomes. I argue that these are a commitment to the neurodiversity paradigm’s concept of disability as difference, and strong versions of the embodiment, embedded and extended theses.

While Robert Chapman’s (2021) ecological functional model is a recent development from the neurodiversity paradigm, both Michelle Maiese’s (2021) enactive reconceptualization of the medical model and Toro et al. (2020) ecological-enactive model are developments from applying enactive and ecological psychology approaches for conceptualizing, investigating and diagnosing disability. Although distinct approaches in themselves, enactivism and ecological psychology both reject assuming minds or cognition are brain-bound, and instead place a strong emphasis on the embodied, intersubjective, and the embedded or socially situated nature of cognitive systems. This commitment is typically presented as a contrast to standard orthodox cognitive science approaches, which are often characterized by methodological individualism where there is a focus on individuals’ internal mechanisms (Chemero and Silberstein, 2008; Gallagher, 2018). In regard to models of disability, Maiese’s (2021) enactive medical model and Toro et al.’s (2020) ecological-enactive model both aim to provide alternatives to the standard medical model of disability by applying enactive and ecological psychology insights for understanding disability.

However, I argue that these models remain problematically committed to an individualistic methodology that leads to a similar kind of undue pathologization of disability that neurodiversity proponents argue is problematic with the standard medical model. This is because for both the enactive medical model and the ecological-enactive model, examining an individual’s capacity for adaptivity is central to determining when a disability should be deemed pathological. While both models explicitly frame themselves as offering a relational model of disability, their strong commitments to an embodiment thesis and the notion of adaptivity lead them to embracing a different version of methodological individualism. In order to reveal how these models remain committed to a form of methodological individualism, I draw on what Kyselo (2014) has labeled the body-social problem, which is the result of a two-horned dilemma that enactivists face when embracing both embodied and embedded claims. By examining Maiese’s (2021) and Toro et al.’s (2020) models’ proposed intervention strategies in the light of the concerns raised by Kyselo’s body-social problem and the neurodiversity paradigm, I show how this theoretical problem becomes a practical problem as well, casting doubt on the efficacy of these models to minimize stigma associated with disability.

After demonstrating the problems that arise with the enactive and ecological models, I present Chapman’s (2021) ecological functional model as a preferable model.^3^ Chapman’s model involves a three-level analysis examining (1) mental traits as they function at the individual level; (2) the contribution of cognitive styles of individuals within their social collectives; and (3) the persistence propensity function or dysfunction as they emerge at the group, ecological level. By utilizing this multi-level analysis, the model not only challenges the pathologization of minority cognitive styles through reframing neurocognitive diversity as a normal and healthy manifestation of biodiversity, but it also offers a fully relational model of disability and a methodology of the kind that the enactive and ecological models aim to offer. I argue that for these reasons, if enactivists want a relational model of disability, then they should adopt both a neurodiversity paradigm approach and Chapman’s ecological functional model.

## 2. Enactivism and ecological psychology

Enactive and ecological psychology approaches to cognition embrace some version of most of the following theoretical assumptions, or theses, regarding minds and cognitive processes, that they are embodied, embedded, enactive and extended, with the last of these being a matter of contention among proponents. Embodiment denotes the claim that cognition is only possible by having a body and that different forms of cognition are shaped by bodily processes and interactions. This often includes the distinction between the “living body,” the body as biologically conceived, and the “lived body,” the body as an experiencing thing. The embedded thesis refers to the assumption that an embodied person is always situated within a particular socio-material environmental setting, and this environmental context shapes the individual’s mind and cognitive processes *via* their embodied engagements with it. The enactive thesis subsumes the assumptions of embodiment and embeddedness, and maintains that cognition and possibilities for action are dependent upon action-perception cycles that are not only shaped by one’s embodiment and embeddedness, but in acting within the world, the individuals enacts their own sense of meaning, or sense-making, that shapes the world to suit their needs and actions. While there are some theorists (Clark and Chalmers, 1998) that only embrace the extended thesis, many enactivists also see this thesis as a part of the enactive approach. The extended thesis posits that cognition can on at least some occasions extend into objects and features of the environment itself, such as in a blind person’s use of cane to navigate their environment or even include other agents when interacting with them (De Jaegher and Di Paolo, 2007; Jurgens and Kirchhoff, 2019).

Proponents of an ecological psychology approach often embrace these aforementioned theses, and utilize the concept of affordances (Gibson, 1977; Chemero, 2003) to explain how the environment provides particular possibilities for action that are shaped by the relationship between a person and their environment (Stilwell and Harman, 2021). Enactivists (Gallagher, 2018; Maiese, 2021) have also drawn on the concept of affordances to explain how an individual recognizes affordances (action-possibilities) within their environment, which are determined by their particular form of embodiment and the bodily skills and abilities they have developed over time (Gibson, 1979; Stoffregen, 2003; Chemero, 2009; Rietveld and Kiverstein, 2014; Toro et al., 2020). Essentially, across both enactive and ecological psychology approaches, there is a shared commitment that in order to explain cognition, action, and behavior, we have to examine an individual’s particular form of embodiment in order to determine what stimuli in the environment the individual is sensitive and responsive to (Maiese, 2018). Importantly, in regard to these various theses, what matters is what aspects of an individual’s body and world play not just a casual or contributory role in shaping their minds and cognitive processes, but what plays a stronger constitutive role.^4^

In regard to the social realm, enactivists utilize the concept of intersubjectivity to explain cognition by examining the salience various aspects of the socio-material world have for an individual, and how the individual interacts with these worldly phenomena. In this sense, enactivism rejects the orthodox approach of neurocentrism, which can be described as a “narrow” perspective on cognition, where cognition is essentially reducible down to brain-based representational processes and contents (Gallagher, 2018). Enactivists and ecological psychologists reject this kind of narrow approach in favor of going “wide,” where the unit of explanation is focused on the brain–body-environment.^5^ As a result, enactivism’s emphasis on intersubjectivity brings to the forefront of investigations of cognition first-person perspectives that, in turn, are able to help explain the normative effects social practices and institutions have on individuals’ cognition and personal identity. For enactivism, intersubjectivity is used to help explain how individuals’ personal identities are constituted through their relationships to the world in regard to self-image, self-esteem, individuality, and social position within society.

By going wide, enactivists and ecological psychologists explicitly reject methodological individualism, the focus on understanding cognition and behavior by focusing primarily on the “cognitive capabilities or mechanisms located in an individual subject, or on processes that take place inside an individual brain” (Gallagher, 2022, p. 160). By embracing the various e-assumptions, they maintain that the world, and the meaning an individual finds within it, is not pre-given or predefined, but is structured by the individual’s embodiment and embeddedness. In this sense, cognitive processes acquire meaning through their role in the context of action, as opposed to an internal brain-bound representational mapping or modeling of the world. These approaches often appeal to dynamical systems theory in order to map and emphasize the relevance of dynamical coupling and coordination across the brain–body-environment relationship. This means that in contrast to the orthodox narrow neurocentric approaches where cognition is explained through appeal to internal mechanisms, enactivists and ecological psychologists emphasize the relational, intersubjective, and socially situated nature of cognition (Gallagher, 2017). Thus, through appealing to notions such as of intersubjectivity and affordances, and through their rejection of narrow brain-bound approaches, enactive and ecological psychology approaches to cognition aim to offer an alternative wide, relational account of cognition that fundamentally rejects methodological individualism.

## 3. The body-social problem

While enactivists have often rejected methodological individualism on the grounds that it implies a problematic dichotomy between brain-bound individuals and the outside world, Kyselo (2014) argues that enactivists are in danger of a new form of methodological individualism related to a dichotomy between body-bound individuals and the outside world. She labels this new form of methodological individualism as the body-social problem, which results from being stuck on one side of a two-horned dilemma. On the one horn, by emphasizing the constitutive relationship of social interaction processes for cognitive identity enactivists risk dissolving the individual in the interaction dynamics that, in turn, downplays the role of embodiment. On the other horn, by emphasizing embodiment over embeddedness, enactivists end up positing a dichotomy between the world and body-bound individuals, where role of the environment is regulated to merely providing contributing causes. It is this second horn of the dilemma that results in this different form of methodological individualism, where what really matters is just the neurobiological form of the cognizing agent. Essentially, by emphasizing embodiment over embeddedness, enactivist fall into the body-social problem. Though both Maiese (2021) and Toro et al. (2020) claim to offer a wide model of disability, where embodiment and embeddedness are integrated and cognition is conceived as relational, I argue that both accounts end up stuck on the second horn of Kyselo’s dilemma, re-embracing methodological individualism.

Kyselo (2014) identifies the body-social problem as arising from two developments in the philosophy of cognitive science that are central to enactivism. These are (1) the “embodied turn,” where the assumption that cognition is brain-bound is rejected in favor of the role the whole body plays in constituting cognition, and (2) the “interactive turn,” where the socio-material environment of a cognizing agent is recognized as playing a constitutive role for its cognitive processes. These developments within enactivism is meant to highlight the importance of social interaction in understanding the ontogenesis of cognition, especially social cognition (Trevarthen and Aitken, 2001; Reddy, 2003; Rochat et al., 2009; Jurgens, 2021, 2022), and in diagnosing and investigating disability and neurodiversity (De Jaegher, 2013; Krueger and Maiese, 2018; Jurgens, 2020; Toro et al., 2020; Maiese, 2021). Both the embodied turn and the interactive turn are attempts to re-determine the boundaries of the individual and entail claims regarding “what counts as the individual (agent, system, person, self) as a whole, each specifying an individuating principle or the essential or minimal sense of this whole” (Kyselo, 2014, p. 3). Yet, Kyselo argues that this individuating principle cannot be both embodied and social, rather it must be either embodied or social, but embracing either of these positions then leads to one of the two horns of the dilemma. Thus, there is a problem for enactivists regarding to how bodily and socio-material environmental aspects figure into the individuation of an individual and their cognitive processes.^6^

As an example of how this body-social problem arises within enactivist accounts, Kyselo (2014) examines participatory sense-making. Participatory sense-making maintains that social cognitive processes are essentially normative, relational, and irreducible to the individual participants of an interaction (De Jaegher and Di Paolo, 2007). On this account, individual participants in a social interaction are regulated in a coupling that constitutes an emergent autonomous organization, where the interactors have to adapt to the external norms that regulate and partly determine the interaction. Kyselo (2014, p. 7) argues that if this is the case, “then the individual would actually not be autonomous but rather heteronomous,” as the individual is not governed by their own laws of self-organization, but is governed by the normatively structured socio-material environment. Yet, this would result in the individual dissolving because they would merge with the socio-material environment rather than emerge from it. De Jaegher and Di Paolo avoid this worry by maintaining that the individual does not dissolve in the interaction because the individual is embodied, claiming that:

“When we speak about cognitive agents in interaction, the basis for such a coupling can take various shapes and involve various perceptual systems, sensorimotor flows, neural, and physiological processes, external objects, and technological mediation. [And that co-regulation involves] bodily variables, such as relative positions and timing between movements, coordination between perceptual systems, and neuro-physiological variables” (De Jaegher and Di Paolo, 2007, p. 492).

Kyselo argues that these strong embodied claims move and position participatory sense-making from the first horn of the dilemma to the second horn, embracing a body-social individualist methodology. Based on these claims, the body, “while differentiating the individual from others, would be a locus of isolation, not a means of connection and engagement” (Kyselo, 2014, p. 7). Kyselo goes on to argue that participatory sense-making is firmly stuck in the dilemma as any attempt to avoid this second horn of the dilemma, leads to the first. Even if proponents of participatory sense-making admit that “individuation of human identity is not fully determined in terms of bodies in isolation,” but instead requires bodily engagements “in socially mediated interactions in the world” this would then lead back to the first horn of the dilemma (p. 7).

Using this example of participatory sense-making, Kyselo concludes that when it comes to defining the individual and the constitutive basis of cognition, enactive approaches often give an ambiguous answer. While explicitly committing to both the embodied and interactive turns, enactivism risks being stuck on either of the two horns of the dilemma where either the bodily or socio-material environmental claims become trivialized in preference for the other. Facing a clearer threat from trivializing the body, enactivists tend to commit more strongly to embodiment, which then leads to a trivializing of the environment and implicitly embracing a methodological individualist position where there is a split between the socio-material world and body-bound individuals.^7^

For the reasons that Kyselo (2014) raises, simply embracing a strong version of embodiment is not enough to move beyond methodological individualism. Essentially, the difficulty with the dilemma is that for accounts that want to embrace both the embodied and interactive turns, depending on how they formulate an individuating principle, they run the risk of trivializing either embodiment or the socio-material world within which the agent is embedded. Supplementing Kyselo’s concern, I argue that while enactivism is meant to offer an integrated account of the body-social through its use of the distinction between the living body and the lived body, its roots in biology, and its appeal to individual adaptivity, leave most versions of enactivism firmly rooted on the embodiment side the dilemma. This means that enactivists often have the tendency to implicitly embrace this body-social form of methodological individualism, under-examining the constitutive influences of the socio-material world.

## 4. The enactive medical model

Maiese (2021) argues that instead of rejecting a medical model approach, we should rather revise the model using insights from enactivism. Her motivation for revising the medical model stems from a desire to avoid an anti-psychiatry stance, which she sees as a social-constructivist view that “downplays and obscures the very real difficulties encountered by subjects with mental disorder” (Maiese, 2021, p. 962). Essentially, her goal is to reconceptualize the medial model so that it treads a middle path between what she labels as the social-construction position of the anti-psychiatry movement, which we can consider as the first horn of Kyselo’s (2014) dilemma, and the purely biologically position of the standard medical model approach. Importantly, like Boorse’s (1975, 2010) standard medical model, the enactive medical model does not attempt to disentangle the notions of disability, pathology and dysfunction, treating disability as inherently dysfunctional and pathological. Additionally, Maiese (2021) wants to remain committed to the medical model’s goal of finding an objective basis for what qualifies as a dysfunctional disability, but having an objective basis that takes seriously the cultural normative influences on how we understand the meaning and significance of the kind of disruptions that make it difficult for individuals to “adapt, live well, and engage effectively with their surroundings” (Maiese, 2021, p. 963).

Maiese’s reconceptualization of medical model focuses on how we can identify and understand disorders as a disruption to sense-making in that the disruption makes it difficult for the individual to “engage effectively with relevant affordance in the surrounding world, including the social world” (Maiese, 2021, p. 963). By conceiving of individuals as complex dynamic systems that are self-organizing, self-regulating, and adaptive, Maiese argues her reconceptualization is a wide, relational approach that accounts for the normative dimension of disorders and takes seriously the biological, psychological, social, and existential aspects of disorders. She maintains that such a wide, relational approach is necessary as many mental, affective, and behavioral problems cannot be reduced to simply physiological terms, but instead, are as Elkins (2009, p. 71) states, “difficult human experiences brought on by faulty learning, inadequate coping skills, stressful events, or other problems in the personal and interpersonal arenas of life.” She applies enactivist conceptions of autonomy, adaptivity and sense-making to (1) account for the normative aspect of disorders and (2) understand the integration of the neurobiological, social, and existential dimensions of disorders.

The notion of autonomy is utilized by enactivists to define cognitive organisms as autonomous systems that are “composed of several processes that actively generate and sustain an identity under precarious circumstances” (Di Paolo, 2009, p. 15), which is derived from Maturana and Varela’s (1980) autopoietic account of a living systems. In this sense, cognition is defined as a living system’s capacity to maintain itself as a recursively self-sustaining network within an environment that requires constant adaptivity. The adaptivity of the system requires it to be able “to define its own identity and distinguish itself from the environment, while at the same time remaining open to material and energetic exchanges with its surroundings” (Maiese, 2021, p. 968). As the system acts in its environment to adapt to changes and sustain its autonomy, its actions change the environment to better suit its own continuity. In this sense, there is an enactive, reciprocal causal (potentially constitutive) relationship between the environment and the cognizing system. The concept of sense-making is then used to explain how a cognizing system is intentionally directed towards its environment, and how normativity (meaning) arises in its perceptions of, and actions in, the world.

Enactivists (Barandiaran, 2017; Di Paolo et al., 2017; Maiese, 2021) claim that through repeated engagements with the world where an individual is making sense of it in order to persist, they develop particular habits, patterns of behavior and attention. Maiese (2021, p. 972) expands on this arguing that established habits in relation to specific socio-cultural environments lead to the creation of ‘regional identities’, which in themselves “gives rise to a new level of normativity that guides perception and action.” The examples of regional identities that she provides include things such as family relationships, friendships, pursuing hobbies, and career success. This new level of normativity relating to regional identities concerns how well an individual fairs and effectively acts within their socio-cultural environment, and is essentially a form of socio-cultural adaptivity.

Centralizing this conception of adaptivity, she argues that functional and adaptive interactions with one’s socio-cultural environment contribute to the self-maintenance of the individual’s particular regional identities and their identity as a whole, while dysfunctional interactions cause the individual’s regional identities to destabilize. This follows de Haan’s (2017) account, as Maiese (2021, pp. 972–973) argues that we should understand “the proximate or immediate cause of mental disorder as a disordered pattern of sense-making” that leads to the destabilization of both an individual’s regional identities and their identity as a whole. Thus, according to the enactive medical model, the objective basis for qualifying disability as pathological or dysfunctional is how a disability harmfully impacts a person’s regional identities leading to an identity to destabilize. This is different from Boorse’s (1975, 2010) traditional medical model that only conceptualizes functioning in strictly biological terms.

Essentially, for Maiese’s (2021, p. 974) enactive medical model, pathological mental disorders consist of disordered patterns of sense-making that are maladaptive, such that an individual’s “engagement and responsiveness to the world is distorted.” Here, pathological mental disorders are understood as an individual being unable to gauge how to regulate their coupling with their environment in order to adapt to changes in the environment and effectively engage with relevant socio-cultural affordances (action-possibilities). She presents her model as a further development of Nielsen and Ward’s (2019) conception of a dysfunctional mental disorder as a “significant and continued violation of an organism’s ‘functional norms,’ one which threatens its organizational autonomy” (Maiese, 2021, p. 974). On both of these models, a functional norm is understood differently from norms based on species typicality (Boorse, 1975, 2010) or evolutionary function (Wakefield, 2007). Rather, this conception of functional norms is centered on self-maintenance in the face of the need for environmental adaptivity. Maiese adds to Nielsen and Ward’s account by arguing that this is essentially a disruption of sense-making, which can also be understood as the inability to engage effectively with available socio-cultural affordances.

For example, Maiese (2021, p. 974) describes depression as when an individual “exhibits heightened attunement to negative features of herself and her surroundings world and many available [socio-cultural affordances] appear to be closed off.” In this case, the depressive individual’s patterns of interaction are too rigid such that it is difficult for her to adjust her sense-making when responding to situational factors. These disruptions to her sense-making capacities can then be considered maladaptive and dysfunctional in that they result in the destabilization of some of her regional identities, or her sense of self as a whole. In this sense, the enactive medical model maintains that a pathologically disabled depressive individual is one for whom numerous affordances no longer appear as salient options. She contrasts this with autism, where she claims there is a loss of particular kinds of affordances relating to engaging with others.

Additionally, she argues that while these examples show how the loss of affordances can determine disorder, she points to schizophrenia as a disorder where an individual’s inability to screen out potentially irrelevant affordances is impaired, leading to behaviors such as so-called “word salad” where the individual engages in producing a jumble of incoherent speech. She claims that what is shared across the examples is “a diminished capacity for engaging with relevant affordances makes it difficult for subjects to fulfill their roles as spouses, friends, workers, colleagues, and community members” (Maiese, 2021, p. 977). Maiese (p. 977) concludes that these kinds of disruptions to an individual’s “patterns of engagement and attention lead to distorted patterns of thinking, feeling, or behaving, which, in turn, render subjects less capable of effective agency.”^8^

Fundamentally, at the core of the enactive medical model is the claim that the objective basis of dysfunctional mental disorders is disordered sense-making due to an inability to adapt to the socio-material environment that results in a destabilization of an individual’s regional identities. This can be identified when a person is “unable to do what they are supposed to be doing, even as what they are supposed to be doing is defined partly in relation to social expectations or their own standards” (Maiese, 2021, p. 980). Importantly, this puts an individual’s capacity for adaptivity at the center of the model, as the objective basis for identifying pathology or dysfunction.

Maiese (2021, p. 981) argues that the enactive medical model is a wide, relational model of disability as even though “enactivism puts the living body at center stage” because “mind is in life” in the sense that “the psychological is inseparable from the biological,” and as such, “mental and social functioning are fully bound up with neurobiological functioning.” In this way, “cognition and experience are best seen as two aspects of the same process,” which is the “embodied action of the living organism within its world” (p. 981). In other words, the model maintains that subjective experience, agency and cognition are all conceived as being physically grounded in the “endogenous process and self-organizing neurobiological dynamics of [individuals’] living bodies as they interact with the world” (p. 981). On this formulation of disorder, it is not just the individual that matters, but the individual’s relation to her surrounding socio-material environment.

Nevertheless, while Maiese presents her model in wide, relational terms embracing the embodiment, embeddedness, and enactive theses, she explicitly rejects endorsing the extended thesis. Citing Hoffman’s (2016) argument for understanding disabilities as extended phenomenon, Maiese (2021, p. 982) claims that “we need not suppose that elements of the environment partially constitute mental health or dysfunction.” Rather, Maiese endorses a version of the embodiment thesis that where the environment is conceived of just playing a crucial role supporting cognitive processes through “complex cognition-sustaining interactions between organism and environment” (Rupert, 2004, p. 396). As Maiese (2021, p. 982) herself writes:

“In my view, the claim that the mind is socially embedded is not only less controversial, but also more consistent with the enactivist approach outlined here. This is because enactivism emphasizes that self-maintenance depends on a process of continual separation and differentiation between organism and environment (Maiese, 2019).”

Although she rejects Hoffman’s extended thesis in favor of Rupert’s embodiment thesis, Maiese maintains that the embodiment thesis leads to the same conclusion as Hoffman’s, which is that the social environment should be a “target of therapeutic interventions” (Hoffman, 2016, p. 1169; cited in Maiese, 2021, p. 982).

Maiese provides a couple of examples for how she conceptualizes these kinds of social environment intervention strategies. She suggests that music therapy can be used to improve autistic individuals’ social cognitive abilities by acting “as scaffolding for [their] ability to gauge fine-grained social cues and engage in ‘body-reading’” (Maiese, 2021, p. 982). She also provides the example of dance-movement therapy as a treatment for depression, as this form of therapy utilizes the “physical layout of the space, the positioning of the participants, props, and particular movement sequences together to help to ‘jump start’ motivation and afford interactive possibilities” (p. 983). These kinds of intervention approaches are meant to change the relationship the individual has with their environment in order improve their adaptability, to re-ordered their sense-making and re-stabilize their regional identities, which should result in an overall improvement to their well-being.

To summarize, Maiese’s reconceptualized enactive medical model maintains that we need to examine the dynamics of an individual’s complex bodily composition and reflexive relationships to their socio-cultural world in order to understand the nature of their mental disorder. In such an analysis, her methodology is to investigate the individual’s capacity for adaptive functioning, as she claims this can clarify the kind of disorder they may have, and the impact it is having on their overall well-being. For such an investigation she argues that the enactivist notions of sense-making, autonomy, and adaptivity can clarify any dysfunctional impacts an individual might have in terms of their affordances and regional identities. The model is offered as a wide, relational approach to disorders and dysfunction, and she concludes that suggests the need for therapeutic approaches need to engage “all the different dimensions of the living person in interaction with the surrounding world” (Maiese, 2021, p. 984).

However, although Maiese is endorsing a version of the embedded thesis within her model, it is a weak version of the thesis. If we consider the enactive medical model in terms of Kyselo’s (2014) dilemma, it is firmly stuck on the second horn of the dilemma as it emphasizes embodiment over embeddedness in centralizing individual adaptivity as the objective basis of disability. In this sense, Maiese’s version of enactivism and her model of disability rely on the body-social form of methodological individualism. As Kyselo argues, this leads to a problematic theoretical inconsistency within the model. Additionally, two other practical issues arise from this individualistic methodology. The first is that like the standard medical model, the enactive medical model equates the disability associated with autism with pathology and dysfunction. Assuming proponents of the neurodiversity paradigm are correct in their criticism of the standard medical model as perpetuating undue pathologization of disorders such as autism, then as Chapman and Carel (2022) argue, this unnecessary assumption may lead to epistemic injustice against disabled individuals in terms of both testimonial and hermeneutical injustice (Fricker, 2008).^9^ This is because when disabilities are assumed to be inherently dysfunctional, they also become stigmatized, which can lead to epistemic injustice that can produce real harms within medical, clinical and research contexts for disabled individuals.

Second, by centralizing an individual’s capacity for adaptivity to determine disability and dysfunction the enactive medical model would determine that an autistic individual’s inability to adapt to neurotypical norms is in itself dysfunctional, regardless if adapting to these norms would be beneficial or harmful for the autistic individual. However, the implication that autistic individuals should align themselves to a neurotypical dominated socio-material environment may in itself create another dysfunction, such as depression, as the individuals may have existential crises in struggling to live up to their culture’s expectations, or their own expectations, which will in itself be shaped by their culture’s standards.

Similarly, if a depressive individual is undergoing an existential crises, this may be due more to features of the environment, such as experiences of economic insecurity, racism, sexism, etc., than any feature of their autonomous individuality and their own capacity to adapt to a harmful environment. In such cases, interventions on the individual may offer some relief for them and increase their adaptivity to their disabling environment, but this would be akin to treating the symptom rather than the actual problem. Instead, interventions need to also be targeted at the system level, and a relational model of disability should have this as a centralized aspect of its approach, even if change on this level is daunting or difficult to achieve.

Essentially, by focusing on an individual’s adaptivity to the detriment of considering how features of the socio-material environment may be producing the dysfunctional disability, we may inclined to focus on intervening on the individual as opposed to the environment that is producing the dysfunction. Maiese’s own examples of social environment interventions are formulated in this way, as they aim at intervening on individuals in order to improve their relational, adaptive capacities with their environments. This is different from interventions that aim at changing the socio-material environment to be more easily adaptable for disabled individuals. As a final example, assuming that Milton’s (2012) argument that many of the social difficulties autistic individuals face result from a problems with neurotypical individuals in understanding autistic individuals, then we have a significant reason to direct interventions beyond just autistic individuals themselves. Instead, we have a reason to intervene on the environment, including neurotypical individuals and neurotypical social practices and institutions, as these aspects of the environment set the norms that a disabled individual, on the enactive medical model should adapt to. Thus, taking these problems into consideration, we can see how an individualistic methodology is potentially harmful for disabled individuals both in terms of epistemic injustice and intervention strategies that may result from even an enactive medical model that aims to provide a wide, relational account of disability.

## 5. The ecological-enactive model

Unlike Maiese’s (2021) enactive medical model, the ecological-enactive model proposed by Toro et al. (2020) is presented as a more radical break from the medical model. As opposed to both the standard medical model and the enactive medical model, the ecological-enactive model has the explicit aim of disentangling the concepts of disability and pathology. Nevertheless, like the enactive medical model, the ecological-enactive model attempts to provide a middle way approach between the medical and social models of disability. However, the model is formulated on taking seriously the lived body and the first-person experience of disabled individuals. In this way, the ecological-enactive model appears at first to align more closely with a social model approach in that it foregrounds “the marginalization, exclusion and oppression of disabled people from full participation in wider society,” focusing on conceptualizing and understanding disability and pathology through phenomenological examinations “how the disabled person experiences the world through their embodiment in it” (Toro et al., 2020, p. 2).

The ecological-enactive model aims to avoid pathologizing the disabled person’s living body by distinguishing between pathology and disability through clarifying the difference between “bodily impairments that are normal, and those that are disabling” (Toro et al., 2020, p. 4). Yet, similar to the enactive medical model, the ecological-enactive model puts an individual’s ability to adapt to changes in their socio-material environment at the center of the account as the principle determining whether a particular disabled individual’s embodiment is pathological. As they say, we need to “understand the distinction between normality and pathology in relation to an individual organism and its capacity to adapt to its environment” (Toro et al., 2020, pp. 5–6), including its socio-material environment. As the enactive approach, with its basis in autopoiesis, is a foundation for this model, it is unsurprising that adaptivity plays a central role in the model’s method of distinguishing between disability and pathology. This is because, as we saw with the enactive medical model, for enactivists, cognition is generally conceptualized as an organism’s ability to regulate its actions in order to maintain itself in the precarious environment within which it is embedded. For human beings, this means being able to not only meet basic metabolic survival conditions, but also social survival conditions. For these reasons, the ecological-enactive model labels this capacity to meet these needs as ‘bodily normativity’ (Toro et al., 2020, p. 6).

Bodily normativity is then used to explain how pathological embodiment can emerge from socio-material practices that “make it too hard or impossible for the disabled person to explore, and establish her own skilled ways of engaging with the relevant affordances” (Toro et al., 2020, p. 13), which includes social affordances that arise in interactions with others. Nevertheless, the model holds that socio-material environments that are “friendly, supportive and flexible” can prevent a disabled individual from being pathologically embodied. In this sense, an individual may have a form of disabled embodiment, but not necessarily be pathologically embodied. Thus, the relationship between disabled embodiment and pathological embodiment is determined by the individual’s capacity to adapt to the socio-material environment within which they are embedded.

To clarify how to distinguish between disabled and pathological embodiment, the ecological-enactive model draws on Canguilhem’s (1991/2015) claim that a non-pathological individual is able to adapt to changes in their environment, and in essence able to follow new socio-cultural norms that they encounter. In this sense, Canguilhem and the ecological-enactive model use the phrase ‘more than normal’ to identify a healthy living body that is capable of adapting to changing conditions by being able to institute new norms in a changing environment when confronted with novel situations. To explain this, Toro et al. provide Canguilhem’s example of an organism that is forced to resettle at a higher altitude, and depending on whether the organism is capable of adapting the changes in this new environment, such as oxygen concentrations, different food, ambient temperature, etc., determines whether “the organism would go from a normal state in the previous environment to a pathological state in the new one” (Toro et al., 2020, p. 7). If the organism is unable to adapt to these new environmental norms, then the organism’s embodiment is classified as pathological. Thus, on this account a normal living body is one that is able to maintain its dynamic stability within a changing precarious environment, even if it is a disabled body. On the other hand, a pathological living body is one that is unable to adapt to maintain its dynamic stability when encountering changes in its environment.

Regarding the relationship between disorder and pathology, Toro et al. hypothesize that if an individual has a pathological embodiment, then this, in turn, can lead to additional mental disorders such as anxiety, which, in turn, can lead the individual to adopting avoidance behaviors in order to “keep everything in the environment as stable as possible, and avoid at all costs unfamiliar things and events” (Toro et al., 2020, p. 8). As a specific example of this, they refer to Goldstein’s (1940) reports on how his patients would avoid taking walks because these could lead to unexpected encounters that would produce catastrophic reactions. This avoidance behavior then has the follow-on effect of a further shrinkage of the individual’s affordances (action-possibilities). Drawing on this, Toro et al. (2020, p. 8) claim that a pathological living body typically only achieves a state of dynamic stability by “arranging their affairs so as to keep the environment as constant as possible at the cost of explorative engagement with the world” that would typically lead to the development of new skills and the opening of new affordances. In this sense, the ecological-enactive model maintains that a decreased capacity for adaptivity due to disability can lead to not only the disability becoming pathological, but also additional pathological disabilities that reduce adaptivity further. Thus, on this model, pathology is characterized by “a stagnation of life in which the person restricts their engagement with the environment with the aim of avoiding catastrophic reactions” (p. 8).

Having developed the conceptual basis of the ecological-enactive model, Toro et al. then conducted phenomenological interviews with individuals with cerebral palsy in order to test the model. One of the interviews involves SG relating her experience of shaking hands, and how she had adapted the practice of shaking with her left hand, as shaking with her right was difficult because of her cerebral palsy. Toro et al. explain her experience stating that “SG did not need to come up with a completely new pattern of social engagement. She just needed to be open to a nonstandard, unconventional way of doing things” (Toro et al., 2020, p. 9). According to the ecological-enactive model, SG may have a disability, but she also has a normal embodiment as opposed to a pathological embodiment because SG is “constantly looking for better ways to perform the exercise,” and even though she might sometimes fail to adapt to changes in her environment, “she feels she can keep looking for better ways to perform an activity” (p. 9). Toro et al. remark that this is different from another interviewee, Michael, who they describe has having a pathological embodiment because of his cerebral palsy as he does not have this kind of flexibility and adaptability when confronted with challenges. Instead, Michael suffers from severe anxiety because of his cerebral palsy that, in turn, leads him to adopt avoidance behavior in order to prevent having to face these kinds of challenges. Thus, while both interviewees have cerebral palsy, according to the ecological-enactive model only Michael’s embodiment is pathological as he is restricted in terms of his affordances (action-possibilities) and capacity for adaptivity.

From their analysis of a variety of interviews, Toro et al. identify two features that distinguish normal from pathological forms of embodiment regarding people with cerebral palsy. They claim that normal embodiment occurs when individuals with cerebral palsy have (1) a preserved capacity for adaptation with novel affordances and (2) are “ready to test established patterns of activity to the best of their ability when circumstances call for them to do so” (Toro et al., 2020, p. 10). This leads them to claim that for disabled individuals, “it is very important to explore for new improved ways of doing things … as well as having a practical knowledge of her own bodily capabilities, skills, and limitations” (Toro et al., 2020, p. 10). Nevertheless, in agreement with a social model approach, Toro et al. argue that social norms inhibit and limit disabled people through the epistemic injustice and oppression they face on a daily basis. As such, though the model conceives of disability as a form of self-experience, it maintains that disability is also intimately bound to an individual’s experiences of, and engagements with, the social world.

For this reason, they end their presentation of the ecological-enactive model by highlighting the importance of the structure of socio-material environments in determining how easy it will be for disabled individuals to adapt to them. As they write, “if the socio-material environment is built around only able-bodied people, [disabled individuals’] practical engagement with the world will become much harder and the risk of becoming pathologically embodied will increase” (Toro et al., 2020, p. 13). In this sense, it is not only the actual physical capabilities of the individual that determines their adaptability, but also “psychological, social and environmental factors that can encourage or discourage them to make the effort and tend toward an optimal grip” (p. 12).

To summarize, the ecological-enactive model maintains that pathological embodiment is not necessarily entailed by disability. Rather, by examining an individual’s embodiment utilizing the tools and methodology of ecological psychology and enactivism it is possible to distinguish between pathological and normal forms of embodiment. At the same time, this kind of examination can do justice to the lived experience of disability through employing phenomenological analyses and interviews with disabled individuals. Nevertheless, what matters for the determination of pathology is whether a disabled individual is able to adequately adapt to their socio-material environment.

However, a similar problem arises here as it did for the enactive medical model regarding the idea that the onus is on the individual to be adaptive. In claiming that disabled individuals need to ‘make the effort’ to be adaptive to their environment the model remains too heavily focused on the individual. While Toro et al. do emphasize the role that the environment can play in inhibiting or limiting a disabled individual’s adaptivity, what distinguishes SG from Michael on their model is that SG ‘makes the effort’ to try new things, to violate established social norms. Only through the others’ acceptance of SG’s use of the left hand is her disability normalized, if the other rejected her change in convention, then she would not have successfully adapted to her environment. In this sense, we can again see that there is too narrow of a focus on individual adaptivity.

Essentially, by relying on the notions of autonomy and adaptivity, enactivist models of disorder risk being stuck on the second horn of Kyselo’s (2014) dilemma in adopting the social-body form of methodological individualism. In such a case, the contributions of the socio-material environment are under considered in terms of their effects on disability. This then leads to two problems. First, it creates a conceptual inconsistency in their accounts regarding their commitments to the embodiment and the embeddedness theses and a wide, relational model of disability. Second, it leads these accounts to putting the onus on the individual for managing their disability, as whether or not their disability is pathological and dysfunction will be determined by the individual’s capacity to adapt to their environment. This second problem could, in turn, potentially lead to undue pathologization of disability and intervention strategies too heavily aimed at the individual, as opposed to the social level. As a solution to these problems in the next section I present Chapman’s (2021) ecological functional account, which I argue is able to escape both horns of the dilemma and offer a truly wide, relational account of disability that avoids undue pathologization of disability.

## 6. The ecological functional model

As Chapman (2021) emphasizes, medical theory and practice is increasingly moving toward approaches that consider more than just the individual, and individual physiology. Instead, theories and practices are refocusing on the health of groups or populations (Arah, 2009; Valles, 2018; Delehanty, 2019). In regard to psychological health, group therapy and systems therapy focus on the family or social group, as opposed to the individual, for diagnosis and treatment. Similarly, Chapman points to accounts of cognitive disability (Hoffman, 2016; Drayson and Clarke, 2020) that are increasingly focusing on how minds are embedded, scaffolded, and extended rather than simply focusing on individuals’ neurobiology. Extending on these developments, Chapman argues that models focusing solely on psychological and physiological individualism are outdated and cannot capture the complex relationship between society and biology.^10^

Working within the neurodiversity paradigm Chapman’s (2021) ecological functional models seeks to provide an account that challenges the pathologizing of minority cognitive styles, arguing that neurocognitive diversity be reframed as normal and healthy manifestations of biodiversity. Nevertheless, central to Chapman’s approach is being able to distinguish between neurocognitive diversity that is normal and healthy, and still be able to identify when, and how, disability can arise for neurodivergent individuals. The ecological functional model does this by examining the relational contributions neurodivergent individuals’ cognitive styles have towards collectives and group functioning alongside examining individual functionality. In doing so, they show that an ecological functional model can provide greater utility for research and intervention strategies than the leading psychiatric functional analyses of mental functions such as Boorse’s (1975, 2010) medical model or Wakefield’s (2007) evolutionary model. In order to avoid interventions that simply target neurodivergent individuals, we need a model that recognizes how disability arises from the relationship between the individual and social levels, as each individual case of neurodivergence has a unique context of factors across both of these levels that need to be investigated and adjusted for in developing interventions strategies. As I’ve argued, this means that more often than is typically proposed, our intervention strategies should focus on the socio-material environment within which a neurodivergent individual is embedded in order to minimize the difficulty of the individual to adapt to challenges and changes within their environment.

In proposing the ecological functional model, one of the Chapman’s aims is to reframe neurodivergent disablement and distress as arising through social barriers and ableist norms, as opposed to centering the model on the cognitive traits associated with a given disability (Chapman, 2019a; Chapman, 2021). While not denying disability, this reframing of disablement as primarily a social and political issue allows the model to emphasize neurodivergent strengths alongside deficits in order to challenge the default pathologization of neurodivergent disability. Drawing on a wide body of research (Murray et al., 2005; Robertson, 2010; Milton, 2012; Chapman, 2019a) utilizing this reframing, the ecological functional model argues that many of the harms associated with disabilities may be better explained by examining external factors in the socio-material world instead of internal individual biological factors. Chapman (2021) highlights that even various critics of social-relational models of disability have accepted that some disabilities that are currently pathologized may be better understood as nonpathological (Jaarsma and Welin, 2012; Wakefield et al., 2020).

Challenging the default pathologization of neurodivergent disability is only one of Chapman’s motivations in developing the ecological functional model. Another motivation is to develop a model in line with the neurodiversity paradigm that is able to avoid charges of relativism (Grinker, 2015) or being anti-science (Costandi, 2019). To do this, Chapman’s (2021) model aims to provide a functional analysis that can account for the claim that neurodivergence itself is a manifestation of healthy natural variation across the species as a whole. Chapman (2021, p. 1361) sees this as a continuation on the foundational claims of the neurodiversity paradigm that has long “emphasized the need to adopt an ecological perspective (Blume, 1998; Singer, 1999; Armstrong, 2015).” As Chapman (p. 1361) points out, Judy Singer, who coined the term neurodiversity, put a specific focus “on how neurodivergent individuals can fill an ‘ecological niche’ (Singer, 1999, p. 66) in society because of their different ways of” experiencing and navigating the world. Similarly, other early and later proponents of neurodiversity, such as Harvey Blume (1998) and Thomas Armstrong (2015), have argued that neurodiversity should be consider akin to cultural diversity and biodiversity in terms of the benefits it can provide to populations as a whole, or ecologies as a whole.

At the center of the ecological functional model is an approach that is based on systems functioning through taking relational and collective functioning into account alongside examining individual functioning, wellbeing, and lived experience. By additionally focusing on relational and collective functioning, this model provides an alternative to both methodological individualism and supposedly objectivist evolutionary functional analysis that underlies both the standard medical model approach (Boorse, 1975, 2010) and evolutionary model approaches (Wakefield, 2007). While the enactive medical model and the ecological enactive model attempt to offer relational approaches for disability, as we have seen both models reliance on the notion of individual adaptivity leads to an implicit embracement of a problematic body-social form of methodological individualism. However, as we’ll see, Chapman’s ecological function model avoid these problems by through its strong commitments to the extended and embedded theses and a multi-level analysis, while still taking into account the intersubjective lived experiences and wellbeing of disabled individuals.

Similar to the enactivist models, the ecological functional model maintains that human cognition is both a product of evolution and a “product of, and sustained by, socioecological scaffolding and is always situated in a broader culture (Oishi and Graham, 2010; Fuchs, 2017)” (Chapman, 2021, p. 1364). Chapman develops the ecological functional model through drawing on Dussault and Bouchard’s (2017) work on ecology where in regard to biodiversity they outline how to understand an organism’s contribution to biodiversity. According to Dussault and Bouchard (2017, p. 1117), an organism’s contribution towards biodiversity is best understood at the system level in terms of the “systems’ ability to thrive and perpetuate themselves in the future,” where an emphasis is placed on how organisms (or species) contribute to the ecosystem’s ability to persist in the face of change. On this view, functions are understood as relational and contextual instead of as intrinsic features of the organism itself. In this sense, the organism cannot be deemed to be functional or dysfunction in itself, function and dysfunction have to be understood in relational and contextual terms in regard to the organism-environment-ecology relationship. While the “propensity of an effect will be intrinsic” to the organism, the function or dysfunction will always be relational and dependent upon the actual behavior of the organism at a given time, and the relation itself can only be understood across the organism-environment-ecology context (Chapman, 2021, p. 1365).

In regard to human mental functions, Chapman argues this emphasis on the ability to persist fits with claims made by neurodiversity proponents, but we can also see how this fits with the claims made by enactivists, such as Maiese (2021) and Toro et al. (2020). First, in regard to the fit with neurodiversity proponents, Chapman states that this aligns with the future orientated claims of neurodiversity proponents, which moves away from the selection history claims made by evolutionary models (Wakefield, 2007). Chapman (2021) provides the example of a report (Aspinal, 2020) by a business on how autistic employees helped the company endure the coronavirus lockdown in 2020 even though this could not have been the reason why the employer hired their autistic employees in the first place. Using this example, Chapman (2021, p. 1365) draws an analogy to how “genetic diversity is adaptive for the propensity of the species to persist given the likelihood of new viruses, regardless of whether the genetic diversity itself was an adaption (i.e., a product of selection).” In this sense, the differences between the autistic and neurotypical employees’ ways of experiencing, navigating and managing the lockdowns meant that the autistic employees provided an ecological advantage for the persistence of the system.

Additionally, Chapman (2021, p. 1365) argues that the ability for an autistic individual to provide greater functional adaptivity for the larger ecological system also fits with the traditional social-relational model of analysis of the neurodiversity paradigm in that their particular ways of being the in world can provide system level benefits. When this possibility is ignored or stifled, such as is the case when “the minority propensity for adaptiveness is often stifled both through a combination of societal disablement and epistemic oppression,” then not only is the disabled individual’s wellbeing harmed, but also dysfunction emerges at the system level as the ecological system in terms of its social and material organization limit its own adaptiveness through its oppressive organization and practices that harm its non-typical members. Here, Chapman provides the example of the prevalent assumption that people with developmental or cognitive disabilities are inherently ineducable. When this assumption is put into practice, these individuals are then often not given access to education, which, in turn, creates an environment in which it is harder for the individuals to either adapt to or contribute to. As Chapman concludes (p. 1365), since an ecological account “can take contingently stifled propensity resulting from oppression or marginalization into account,” it has the benefit of shifting toward “acknowledging contingently stifled propensity,” which, in turn, can help “avoid the reification of social facts” that would result not only in establishing or maintaining oppressive, harming socio-material structures, but also potentially limit the functioning and adaptivity of the ecological system as a whole.

Second, in regard to the fit with claims made by enactivists, this focus on adaptability and system level self-maintenance closely aligns not only with the central focus and claims made by Maiese (2021) and Toro et al. (2020) in their models, but also with the enactive concept of autopoiesis. However, a significant difference between Chapman’s model and these enactivist models is that Chapman is additionally examining functionality at the ecological system level, as opposed to solely the individual level. For the enactivist models adaptivity is conceived of in terms of the individual’s capacity to adapt to the environment, and when the individual is unable to adapt, in a way that leads to harm in terms of limitation on the individual’s affordances (action possibilities) then the individual’s disability is labeled pathological. As Chapman’s focus is on both the individual and ecological adaptive functionality, this same conclusion is not necessarily reached. Instead, if the individual’s inability to adapt to their environment is due to socio-material aspects of the environment that stifle their propensity of adaptability, then the dysfunction that one would label as pathological is due to system level features, not individual level features. Thus, by examining functionality and adaptivity across both the individual and ecological levels, the ecological functional model avoids the body-social problem and any form of methodological individualism.

Chapman (2021, p. 1365) emphasizes this point stating that “in ecology functional roles are multilevel and relational rather than restricted to individuals.” In other words, ecological functional roles are “attributed either when some part of the ecosystem plays a role within biodiversity or ecosystem functioning or when biodiversity itself, or its component parts, plays a role (Nunes-Neto et al., 2014)” (p. 1365). Drawing on the work of other neurodiversity proponents, Chapman identifies three levels of mental functional propensity, which they argue are central to the ecological functional model.

The first level is examining the mental traits of an individual in regard to the persistence propensity of a particular individual (Robertson, 2010), which requires a sensitivity to both the strength and limitations of minority modes of functioning. This level is centered on examining the how a disabled individual’s traits support or hinder both their wellbeing and their interactions with their socio-material environments, similar to the focus that individualist accounts take, but specifically examining particular interactions the individual has with their socio-material environment, and their reported lived experience from these interactions. Additionally, this level requires examining the traits of non-disabled individuals that disabled individuals encounter, as these also in part determine the kinds of interactions and lived experiences the disabled individual has, as for example, communication issues between autistic individuals and neurotypical individuals likely result from a double empathy problem (Milton, 2012). In more detail, by examining an autistic person’s traits on this level the model may be able to determine the extent to which their monotropic attention patterns (Murray et al., 2005) may hinder their interactions with neurotypical individuals with polytropic attention patterns (Milton, 2012; Jurgens, 2020), or how monotropic attention patterns make certain environments uncomfortable for an autistic individual due to too much sensory stimulation. In both of these cases, the model would examine how monotropic attention patterns combined with particular features of an individual’s socio-material environment may harm their wellbeing and their persistence propensity. However, the model also require examining how their monotropic attention patterns may improve their capacity to focus on certain tasks or endeavors or their interactions with other autistic individuals, which may improve their wellbeing and their persistence propensity when it helps them succeed on a given task, simply offer an enjoyable experience or connect with another autistic individual.

The second level involves exploring the contribution of specific cognitive styles, which are collections of traits and patterns of thought and behavior of individuals, within a larger social collective to which they belong, which Chapman (2021, p. 1365) labels as a “niche contribution.” Here the focus is on how specific cognitive styles contribute specific roles in the collective. For example, Chapman points to Blume’s (1998) claim that “cybernetics and computer culture, for example, may favor a somewhat autistic cast of mind” (para. 4; cited in Chapman, 2021, p. 1365). In this sense, specific cognitive styles found among autistic individuals may provide particular advantages for engaging with establish practices within particular social collectives like computer culture, which may both improve the individual’s wellbeing and persistence propensity as they find a collective they thrive in while simultaneously improving the persistence propensity of the collective and other members within it. However, investigations on this level also require examining how specific cognitive styles may be disadvantageous for both the individual and the collective’s persistence propensity, and simultaneously harm the individual’s wellbeing.

The third level requires investigating persistence propensity function or dysfunction as they emerge at the group, ecological level. Utilizing Hoffman’s (2017) work, Chapman (2021, p. 1365) argues that there may be collective cognitive traits that are “more or less adaptive for the collective, in relation to the environment, that emerge from the group rather than being directly traceable to individual members.” In this sense, examinations at this level assume a version of the extended cognition theses where groups of individuals, collectives, can have their own unique cognitive traits that arise through both the interactions of its members with their socio-material environments and the interaction between its members. An example Hoffman (2017, p. 2228) provides is that of a non-diverse company failing to promote its product over the competition’s because “All members of this company’s marketing team have detail-oriented, somewhat creative, but relatively non-divergent and neurotypical ways of thinking” that results in less innovative and novel ways of marketing their product. In this sense, the group’s persistence propensity is harmed by not having any neurodiversity. While the examination at this level is primarily focused on the functioning of the collective, and though this functioning cannot be directly traced to any single member of the collective as how members interact with, and either support or hinder, each other within the collective in part determine the collective’s overall functioning, this level of analysis still requires determining how individuals members’ cognitive styles contribute to (or hinder) the collective’s cognitive functioning. Thus, a central feature of the ecological functional model is that it allows for the possibility that any trait could contribute to relational functions or dysfunctions not only at any of these three levels, but also, that a trait could contribute to both functions and dysfunctions simultaneously.

Chapman (2021, pp. 1365–1366) offers a tentative definition of functional as “a mental trait, cognitive style, or group must have the propensity to perform an effect that contributes to either individual or group persistence, or both.” In contrast, they define dysfunction as an occurrence “whenever there is a relational clash between any of these levels that hinders the propensity to persist” (p. 1366). By defining function and dysfunction across these three levels, (1) mental traits, (2) cognitive styles and (3) group ecology, the ecological functional model maintains that “groups themselves have traits that are functional or dysfunctional in relation to the group or individual persistence” (p. 1366). Thus, unlike the enactivist models (Toro et al., 2020; Maiese, 2021), functionality while being understood as a capacity for persistence (or adaptivity in enactivist terms) is conceived in individual and group relational terms that avoids the pitfalls of the social-body problem and a methodological individualist position. Importantly, this multi-level functional analysis, which includes the group level, embraces strong versions of the extended and embedded claims as functionality and persistence is conceived of in terms of the organism-environment-ecology relationship. In other words, a neurodivergent individual’s mental traits and cognitive style cannot be examined solely in terms of their embodiment and their capacity for adaptivity to their environment, but must also be considered in terms of how their environment facilitates or hinders (1) their individual capacity for adaptivity and wellbeing and (2) their contributions to group adaptivity.

To demonstrate how it’s possible for a neurodivergent individual’s mental traits or cognitive style could be beneficial for individual and group propensity, while also being reconceptualized along ecological niche contribution lines, Chapman again turns to autism as an example. While autism has traditionally been framed in terms of deficiencies, recent empirical studies have shown that autistic traits and cognitive styles can be beneficial as well. Some of these traits include capacities for “hyper-systematizing (Baron-Cohen, 2006; Greenberg et al., 2018), hyperattention to detail (Fitch et al., 2015), intense ability to focus (Murray et al., 2005), and reduced susceptibility to framing effects (De Marinto et al., 2008)” (Chapman, 2021, p. 1366). Chapman argues that through the ecological functional model these traits can be considered as niche contributions in that they improve the functionality for the group persistence propensity.

Importantly, even autistic mental traits that are typically considered as ‘deficits’ could contribute to functions at the group level while still being associated with individual disability. Here, Chapman (2021, p. 1366) provides the example of autistic individuals tending to be less “spontaneously attuned to neurotypical social worlds than neurotypical individuals are (Milton, 2012; Chapman, 2019b),” which while disabling on an individual level in making it harder for these autistic individuals to adapt to the neurotypical social world, can lead to an increase in their originality of thought (Happe and Vital, 2009) and a form of agency that tends to be freer from established social pressures (Baron-Cohen, 2011). This divergent cognitive style has been associated “with a tendency to be ‘super moral’ in terms of following social rules with less restraint from social pressures (Chapman, 2021, p. 1366). Chapman provides the example of autistic climate activist Greta Thunberg, who credits her autism for her success as an activist (Silberman, 2019). We can also consider Grace Tame, named Australian of the year in 2021, an autistic activist survivor who championed changes regarding sexual assault laws in Australia, and who also credits her autism for her success (Tame, 2022). Though their mental traits and cognitive styles are both beneficial and disabling in regard to their individual level, on the ecological level their autistic mental traits and cognitive styles serve a niche-functional role in providing greater group adaptivity.

While defenders of the traditional medical model may argue that the examples provided here only consider a subset of autistic individuals and not those with high support needs, Chapman recognizes this and notes that not all neurodivergent individuals will exhibit strengths associated with their disabling mental traits on either the individual or social level, especially multiply disabled autistic individuals with high support needs. In regard to identifying and understanding dysfunction associated with multiply disabled individuals on the ecological functional model, Chapman (2021) claims that this still must be done in a relational multi-level analysis where (1) individual traits are considered in relation to the way in which the socio-material world is organized and (2) without committing to the additional claim that these individuals, or aspects of their way of being in the world, are intrinsically pathological. This relational multi-level methodology makes it possible to determine how the organization of the socio-material world contributes to dysfunction in relation to each disabled aspect of the individual in terms of hindering both their wellbeing and their propensity for self persistence, i.e., their capacity for adaptivity. Chapman suggests that as the neurodiversity perspective is to view individuals as valuable regardless of their functional propensities, their model would nevertheless provide greater utility in understanding these individuals’ disabilities than deficit-based evolutionary framing models. Finally, while I argue here that the ecological functional model is preferable to either the enactive medical model (Maiese, 2021) or the ecological-enactive model (Toro et al., 2020) in terms of an explicitly relational model, following Chapman I take a pluralistic stance leaving room for the use of the medical model for particular forms of disability, such as infant anencephaly. As Chapman (2021, p. 1367) states, what the ecological functional offers is allowing “the benefits of both social-model and medical-model interventions where appropriate, yet in a way that avoids locating the dysfunction in the individual’s way of being.”

Overall, the aim of the ecological functional model (Chapman, 2021) is to provide a relational model that can serve as a useful basis for directing future scientific research, improving clinical understanding of neurodiversity and improve the public understanding of disability. In terms of scientific research, the model encourages researchers to focus not on the question of “What is wrong with this individual or group in relation to those who are normal?,” but instead focus on the question “How can we understand the strengths, limitations, struggles or potential of this group or individual in the wider social context?” (p. 1368). Regarding clinical understanding, the model allows greater flexibility for psychotherapeutic practice or the development of individual self-understanding as it can examine the complexity of psychological ability and disability in the broader organism-environment-ecology context. Lastly, by shifting way from individual pathology towards relational dysfunction and function the model helps alleviate the stigma surrounding disability, which, in turn, would minimally alter the interpersonal aspects of the socio-material environment facilitating neurodivergent individuals’ adaptability.

## 7. Conclusion

This article has been concerned with how we can move away from examining disability through individualist methodology, and has argued that Chapman’s (2021) ecological functional model offers an account that is able to do this while conceiving of disability and neurodiversity as truly relational phenomenon. As opposed to both Maiese’s (2021) enactive medical model and Toro et al.’s (2020) ecological-enactive model, Chapman’s model does not rely on determining disability by centralizing its investigation on an individual’s capacity to adapt to their environment. Instead, disability is investigated within the relationships the individual has with their socio-material environment across three level of analysis: 1) how mental traits function at the individual level, 2) how cognitive styles contribute to social collectives, and 3) examining persistence propensity function or dysfunction as they emerge at the ecological level. Using this multi-level analysis, disability is not determined solely by the individual’s adaptive capacity, but rather is also determined by the social community’s capacity to facilitate or hinder diverse individuals’ adaptability and well-being, and the contribution a disabled individual is able to provide on the group level.

Although Chapman does not explicitly adopt an enactivist approach in their formulation of the ecological functional model, they do explicitly endorse Hoffman’s (2016, 2017) extended thesis that neurodiversity should be understood in a strong sense to be an extended phenomenon. Additionally, I argue that the account also implicitly endorses a much stronger version of the embeddedness thesis than either of the enactive models considered. In embracing these strong versions of extended and embedded theses and through utilizing a multi-level analysis, I argued that Chapman’s model takes a truly wide, relational approach in its formulation of neurodiversity and disability, which offers a methodology that is not individualistic and avoids the body-social problem as formulated by Kyselo (2014). For these reasons, if enactivists want a relational model of disability, then they should consider adopting both a neurodiversity paradigm approach and Chapman’s ecological functional model.

## Data availability statement

The original contributions presented in the study are included in the article/Supplementary material, further inquiries can be directed to the corresponding author.

## Author contributions

AJ was the sole contributor to the conception and production of the manuscript.

## Conflict of interest

The author declares that the research was conducted in the absence of any commercial or financial relationships that could be construed as a potential conflict of interest.

## Publisher’s note

All claims expressed in this article are solely those of the authors and do not necessarily represent those of their affiliated organizations, or those of the publisher, the editors and the reviewers. Any product that may be evaluated in this article, or claim that may be made by its manufacturer, is not guaranteed or endorsed by the publisher.

## Footnotes

^1^Without denying that having disability often results in greater impacts to one’s wellbeing, this neutral way of conceptualizing disability follows neurodiversity proponents’ aim to leave it an open question as to how much social barriers and ableist norms impact a disabled individual’s wellbeing as opposed to assuming that their disablement and distress is primarily a result of their different traits associated with their given disability (Chapman, 2021).

^2^Nevertheless, neurodiversity proponents also recognize that these differences often do result in additional difficulties or harms for disabled individuals in comparison to ‘normal’ individuals, and as such do not reject using the term ‘disability’.

^3^See Supplementary Table 1 for a general overview of the core commitments of these three relational models of disability.

^4^The term constitution as it is used here, and throughout this article, should be taken as a species of causation, i.e., constitutive causation. Constitution used in this enactive sense is meant to capture the bidirectional aspect of enactive relationships where there is continuous reciprocal causal influence between individuals and their environments (Jurgens and Kirchhoff, 2019).

^5^On these kinds of wide approaches, the brain is not dismissed in attempts to understand cognition; rather, it is de-centered as greater focus is put on the roles of bodily and environmental factors (Gallagher, 2018).

^6^I take this not only as a problem for enactivism, but for any account of cognition that aims to provide a wide, relational approach that simultaneously wants to emphasize both embodiment and embeddedness as constitutive bases of cognition.

^7^Kyselo (2014) raises the dilemma in addressing conceptions of self, and her own solution is formulated in terms of a social version of adaptive regulation. Whether or not her solution is satisfactory for that discussion, or whether it is satisfactory for models of disability is not taken up here. Instead, I argue Chapman’s (2021) ecological functional model avoids the dilemma altogether.

^8^Maiese (2021) clarifies that on her model behavior is not simply pathological because it is not ‘normal’ in terms of social cultural expectations, as this might lead to labeling homosexuality as a disorder. Instead, the behavior needs to be incapacitating in some way.

^9^See also Kidd and Carel (2018, 2019) for extensive discussions on epistemic injustice in medical and clinical contexts.

^10^Nevertheless, as argued in Chapman (2021) and later in this section, the ecological functional model is offered through a pluralistic stance as a relational model of disability, and as such, is not argued to be taken as the only model of disability. As Chapman claims, the medical model may still be the best model for certain forms of disability.
