# Supplementary material for: Body social models of disability: Examining enactive and ecological approaches
Source: Front Psychol. 2023 Mar 8;14:1128772. doi: 10.3389/fpsyg.2023.1128772 (PMC10032405; doi:10.3389/fpsyg.2023.1128772)
Supplement: Supplementary file 1 [file Data_Sheet_1.PDF]

Supplementary Material

Body Social Models of Disability: Examining Enactive and Ecological Approaches

Dr Alan Jurgens*

*** Correspondence:** Dr Alan Jurgens: ajurgens@uow.edu.au

1 Supplementary Data

Supplementary Material should be uploaded separately on submission. Please include any supplementary data, figures and/or tables.

Supplementary material is not typeset so please ensure that all information is clearly presented, the appropriate caption is included in the file and not in the manuscript, and that the style conforms to the rest of the article.

2 Supplementary Figures and Tables

Table 1. Summary and comparison of the core commitments of the three relational models of disability. Note that these are simplified claims and do not fully capture the nuance of the core commitments of the models.

Model of Disability	Core Theoretical Assumptions	Conceptual Relationships	Practical Intervention Recommendations
Enactive Medical Model<br><br>(Maiese, 2021)	Cognition is Embodied & Embedded.	‘Disability’ = ‘Disorder’<br><br>‘Disability’ determined by examining agent’s adaptiveness to their environment.	Primarily targeted at the individual level to improve individuals’ capacity to adapt to their environments.

Ecological-Enactive Model<br><br>(Toro et al., 2020)	Cognition is Embodied, Embedded &<br><br>Extended.	‘Disability’ ≠ ‘Disorder’<br><br>‘Disorder’ determined by examining agent’s adaptiveness to their environment.	Primarily targeted at the individual level to improve individuals’ capacity to adapt to their environments.
Ecological Functional Model<br><br>(Chapman, 2021)	Cognition is Embodied, Embedded &<br><br>Extended.<br><br>The Neurodiversity Paradigm is required to minimize the stigma surrounding disability.	‘Disability’ ≠ ‘Disorder’<br><br>‘Disability’ & ‘Disorder’ determined by individual traits and the aspects of the socio-material environment to facilitate or hinder individuals’ adaptability and well-being.	Primarily targeted at the social level (including non-disabled individuals), but also targeted at the individual level when required.
